# Effects of Circuit Training Program on Cardiovascular Risk Factors, Vascular Inflammatory Markers, and Insulin-like Growth Factor-1 in Elderly Obese Women with Sarcopenia

**DOI:** 10.31083/j.rcm2304134

**Published:** 2022-04-08

**Authors:** Won-Sang Jung, Yae-Young Kim, Jeong-Weon Kim, Hun-Young Park

**Affiliations:** ^1^Physical Activity and Performance Institute (PAPI), Konkuk University, 05029 Seoul, Republic of Korea; ^2^Department of sports medicine and science, graduated school, Konkuk University, 05029 Seoul, Republic of Korea; ^3^Department of oriental sports medicine, Daegu Haany University, 38609 GyeongBuk, Republic of Korea; ^4^Graduate School of Professional Therapy, Gachon University, 13120 Gyeonggi-do, Republic of Korea

**Keywords:** circuit training, cardiovascular risk factors, inflammatory markers, insulin-like growth factor-1, sarcopenic obesity, elderly population

## Abstract

**Background:**

Sarcopenic obesity is caused by a decrease in muscle mass and an increase in body fat due to aging, and has been the cause of cardiovascular diseases such as hypertension, diabetes, hyperlipidemia, and arteriosclerosis and high inflammatory conditions. However, there is a lack of research on the effects of long-term exercise training as regards to the body composition and blood-related physiological indicators. Therefore, the purpose of this study was to investigate the influences the effect of circuit exercise training for 12 weeks on cardiovascular risk factors, vascular inflammatory markers, and insulin-like growth factor-1 (IGF-1) in elderly obesity women with sarcopenia.

**Methods:**

A total of 28 elderly obese Korean women with sarcopenia (75.0 ± 5.1 years) were randomly assigned either to a control group (CG, n = 14) or an exercise group (EG, n = 14). The EG performed circuit exercise training for 25–75 minutes (gradually incremental) three times per week over a period of 12 weeks, while the CG maintained their usual daily lifestyle during the intervention period. Pre- and post-intervention evaluations were performed on selected cardiovascular risk factors, inflammatory markers, and IGF-1.

**Results:**

The EG group exhibited improved body composition (i.e., body mass index, fat-free mass, % fat mass, waist-to-hip ratio; all *p *< 0.030, η^2^ > 0.169), Cardiovascular risks factor (i.e., heart rate, systolic blood pressure, rate pressure product, high-density lipoprotein cholesterol, total cholesterol/HDL-C ratio, triglyceride/HDL-C ratio, low-density lipoprotein cholesterol/HDL-C ratio, brachial-ankle pulse wave velocity, fasting plasma insulin, homeostasis model assessment-insulin resistance; all *p *< 0.042, η^2^ > 0.150), Inflammatory markers (i.e., high sensitivity C-reactive protein, interleukin-6; all *p *< 0.045, η^2^ > 0.146), and IGF-1 (*p* = 0.037, η^2^ = 0.157). Conversely, there were no significant changes observed in CG.

**Conclusions:**

Twelve weeks of circuit training had a positive effect on the improvement in cardiovascular risk factors, vascular inflammatory markers, and IGF-1 in elderly obese women with sarcopenia.

## 1. Introduction

Sarcopenic obesity which is characterized by the increase in body fat, decrease in fat-free mass and muscle strength has been attributed to aging [1]. Sarcopenia symptoms include a decrease in muscle mass, muscular strength and functions associated with age, with the elderly people at high risk of sarcopenic obesity [2, 3]. Sarcopenia is commonly as a result of lack of physical activity, and it is reported that the risk of cardiovascular diseases is increased when sarcopenia is combined with obesity [4]. In addition, fat infiltration in the skeletal muscle worsens insulin resistance [5], and the vicious cycle of reduction in energy consumption and increased risk of falling is as a result of greater sarcopenia and obesity [6]. Moreover, increased intake of high fat diet results in increased cholesterol and triglycerides in the blood, which causes hyperlipidemia [7], and reduced vascular elasticity, which gives rise to arteriosclerosis accompanied by stenosis and obstruction of the vascular wall [8, 9].

A decrease in muscle mass and muscle strength has been reported to act as an independent risk factor for atherosclerosis [10]. Insulin-like growth factor-1 (IGF-1) is also a known independent risk factor of sarcopenic obesity, and the reduction of growth hormone, dehydroepiandrosterone’s (DHEAs), estrogen and testosterone precursor, caused by aging decreases sarcopenic obesity is also known to be a direct cause [11]. Additionally, sarcopenic obesity is closely related to the systemic inflammatory state, and level of inflammatory cytokines, interleukin-6 (IL-6) and C-reactive protein (CRP) [12, 13]. Hence, in older people whose physical function is deteriorated as a result of aging, it is more likely to aggravate various diseases and physiological problems caused by sarcopenic obesity. Consequently, efforts to prevent and improve muscular obesity are urgently required.

Particularly, exercise therapy has been reported to be the most effective way to prevent and remedy sarcopenic obesity [14]. However, single exercise has some limitations in older adults. Aerobic exercises are limited to increasing muscle mass in the elderly [15], and resistance exercise poses a high injury risk while repetitive motion can be monotonous [16]. In order to compensate for these short-comings, the study proposed circuit exercises consisting of a combination of flexibility, aerobic and resistance exercises. Circuit exercise is reportedly an effective way to develop cardiovascular and muscular functions simultaneously owing to its low risk of injury, interesting exercises without being constrained by cost and location, and alternates between aerobic and resistive movements [17]. In particular, circuit training as a fitness program for the elderly was selected as the world’s top health and fitness trend by the American College of Sports Medicine for 2021 and 2022 [18, 19].

As highlighted above, sarcopenic obesity has been causal factor of cardiovascular disease such as hypertension, diabetes, hyperlipidemia, and arteriosclerosis, and high inflammatory status. However, there is a lack of research on the effects of long-term exercise training as regards to the body composition and blood-related physiological indicators. Therefore, we carried out a randomized trial to investigate the influences of circuit exercise training for 12 weeks on cardiovascular risk factors, vascular inflammatory markers, and insulin-like growth factor-1 (IGF-1) in elderly obesity women with sarcopenia.

## 2. Materials and Methods

### 2.1 Participants

The study subjects included 28 elderly obese Korean women with sarcopenia (mean age = 75.0 ± 5.1 years) in community-dwelling. Table 1 presents their physical characteristics. The inclusion criteria were as follows: (1) aged 65 years and above; (2) have sarcopenia comorbid with obesity; (3) should not have performed any kind of exercise over the past six months prior to the study, and (4) willing to fully participate. The exclusion criteria included older adults who: (1) had an ill controlled chronic diseases; (2) had previously undergone retinal laser treatment; (3) had a history of acute myocardial infarction; (4) had joint replacement or fracture of the lower limb six months prior, and (5) had severe cognitive disturbance. The women who met the selection criteria were randomly divided into a circuit exercise training group (EG, n = 14) and a control group (CG, n = 14) using a computerized random number generator.

**Table 1.** **Participants’ characteristics. Data are means (± SD)**.

Variables	CG (n = 14)	EG (n = 14)	*p*
Age (years)	74.64 ± 5.77	75.36 ± 4.50	0.718
Height (cm)	153.01 ± 4.41	152.58 ± 4.30	0.797
Weight kg)	52.91 ± 5.03	52.43 ± 5.20	0.804
BMI (kg/$m^{2}$)	22.58 ± 1.69	22.50 ± 1.75	0.899
Free fat mass (kg)	32.89 ± 2.56	32.51 ± 1.93	0.663
Body fat mass (%)	35.33 ± 3.18	35.10 ± 3.13	0.847
ASM (kg/$m^{2}$)	5.17 ± 0.59	5.27 ± 0.26	0.573

Note: SD, standard deviation; CG, control group; EG, exercise group; BMI, body mass index; ASM, appendicular skeletal mass.

**Fig. 1.**
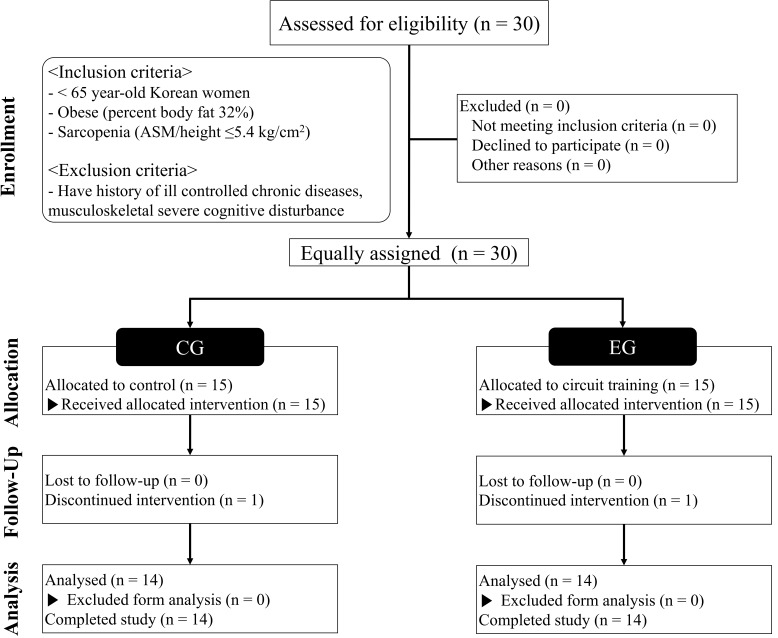
**CONSORT (Consolidated Standards of Reporting Trials) flow diagram**.

Sarcopenia is defined as an appendicular skeletal muscle mass (ASM, kg)/height ($m^{2}$). The determinant threshold value is ≤5.4 kg/$m^{2}$ for women [20]. Obesity assessment criteria define the second highest quintiles of whole-body fat % [21]. Based on the fifth period of the National Health and Nutrition Examination Survey, the study defined the top 40 percentile of body fat rate (% body fat 32%) as obese, and was calculated for 1072 elderly women aged 65 and above. The participants provided a written informed consent after being taken through the experiment and having understood the possible adverse effects. The consolidated standards of reporting trial (CONSORT: Consolidated Standards of Reporting Trials) flow diagram is shown in Fig. 1. This study was approved by the Korean Institutional Review Board (KHSIRB-16-016). All procedures were in accordance with the Helsinki Declaration.

### 2.2 Study Design

Blood sampling and body composition were measured for all participants by pre- and post-test. The EG participants then began following the exercise intervention consisting of 45~75 minutes, three times per week for a total of 12 weeks. The circuit training consisted of 10 movements: walking in place, shoulder press and squat, twist to dash, lunge, jumping jacks, kick back, push up, crunch, hip bridge and bird dog. The program included 10 minutes each for warm-up and cool-down, and the main exercise set was performed for 10 minutes followed by five minutes of rest in between sets. The training session lasted 25 minutes in week 1~2, 40 minutes in weeks 3~8, and 55 minutes in weeks 9~12. The exercise group subjects were monitored by wearing a polar (m400, Polar, Helsinki, Finland) during exercise, and the exercise intensity level was in the range of 60–80% of the heart rate reserve (HRR) (Table 2). To minimize the effect of extrinsic variables on dependent variables, education on dietary intake and daily activities was conducted twice a week. All exercise interventions were conducted at a constant temperature and humidity level of 23 ℃ and 60%.

**Table 2.** **Circuit training program**.

Stage	Phase	Mode/set	Duration	Intensity
Warm-up		Stretching	10 minute	
Main exercise	Phase 1	1∼2 weeks: 2 set	25 minute	HRR 60∼80% 1 set (10 minute) Rest (5 minute) 3 times/week
	Phase 2	3∼8 weeks: 3 set	40 minute	
	Phase 3	9∼12 weess: 4 set	55 minute	
Cool-down		Stretching	10 minute	

In contrast, the Control group participants (CG) did not participate in any exercise intervention. The CG sustained their usual physical activity lifestyle for the duration of the study. In this routine, CG participants were encouraged to check in by telephone twice a week to maintain their daily lifestyle (physical activity and dietary intake), and were taught nutrition education in the lab every four weeks. In order to keep the amount of food intake as constant as possible during the 12-week treatment period, the subjects in all groups were investigated for their daily energy intake using a three-day dietary log (2 days on weekdays and 1 day on weekends). Energy intake was recorded every 4 weeks, and total caloric intake was analyzed using a Computer Aided Nutritional analysis program (CAN-Pro, version 5.0, The Korean Nutrition Society, Seoul, Korea) based on each individual’s meal diary. In addition, in order to evaluate the amount of normal physical activity, the total daily energy consumption (kcal) was measured by wearing the Lifecorder (Kenz, Japan) on the subject’s right waist (the center of the navel and flank glands). It was worn for 5 days excluding weekends, and was worn except for sleeping and showering time. There was no difference between the two groups or time period (Table 3). The CG participants underwent similar pre-intervention and post-intervention testing as EG participants.

**Table 3.** **Energy intake and physical activity. Data are means ( ± SD)**.

Variables	Group	Intervention				*p*-value
		0 week	4 week	8 week	12 week	
Energy intake (kcal/day)	EG	1408.97 ± 497.40	1439.32 ± 514.38	1493.20 ± 109.36	1443.49 ± 396.57	0.432
	CG	1354.86 ± 310.12	1374.21 ± 291.50	1407.23 ± 172.88	1412.71 ± 296.63	0.505
	*p*-value	0.623	0.558	0.671	0.742	
Physical activity (kcal/day)	EG	1721.11 ± 54.15	1728.76 ± 62.19	1832.91 ± 29.85	1810.96 ± 74.72	0.746
	CG	1854.86 ± 310.12	1874.21 ± 291.50	1910.46 ± 30.16	1812.71 ± 296.63	0.861
	*p*-value	0.518	0.672	0.498	0.854	

### 2.3 Measurement

All participants had been fasting for more than 12-hours before coming to the laboratory. Blood samples were collected between AM 8:00 and 9:00, after the subjects rested for 30 minutes. Afterwards, body composition was measured. Blood samples were collected from venous blood (10 mL) by a trained nurse in a sitting position for all subjects. The collected blood was placed in a tube that has with and without heparin according to each analysis item, centrifuged at 3000 rpm at 4 ^∘^C for 15 minutes using a centrifuge, and was separated into plasma and Serum was collected, aliquoted into storage tubes, and stored in a deep freezer at –80 ^∘^C until blood analyses. Frozen samples were thawed at room temperature, and all analyses were tested in duplicate by the same technician on the same day. Body composition parameters (height, weight, BMI, free-fat mass, and percent body fat) of the participants were measured using bioelectrical impedance (InBody770, Inbody Co., Seoul, Korea) and dual-energy X-ray absorptiometry (DEXA, Hologic QDR 4500, Hologic, Bedford, MA, USA) analysis equipment. ASM, a value excluding the bones and fat of the arms and legs was used among the values measured whole body by DEXA [18]. The Waist-to-hip Ratio (WHR) was calculated as waist circumference divided by hip circumference. Waist and hip circumference were measured according to the World Health Organization (WHO) recommendations. The subject was asked to stand relaxed with arms at the sides, feet positioned close together, and weight evenly distributed across feet. Waist circumference measurement was made at the approximate midpoint between the lower margin of the last palpable rib and the top of the iliac crest. Hip circumference measurement was taken around the widest portion of the buttocks. To assess hypertension related variable, the resting pulse rates and blood pressures were measured twice, in the sitting position after a minimum of 5 minutes rest, using an automatic sphygmomanometer (HBP-9020; OMRON Colin, Tokyo, Japan), and the average value was used for analysis, and mean arterial blood pressure (MAP = DBP + (SBP – DBP)/3), pulse pressure (PP = SBP – DBP), and rate pressure product (RPP = HR × SBP) were calculated [22]. Arteriosclerosis related variables, such as TC/HDL-C ratio, TG/HDL-C ratio, and LDL-C/HDL-C ratio were calculated using hyperlipidemia related variables [23]. The arterial stiffness was measured using automated VP-1000 plus (Omron, Kyoto, Japan). Measurements were performed wearing pressure cuffs on the subject’s arms and ankles and after the subject rested in the supine position for approximately 10 minutes. The average of right and left brachial-ankle pulse wave velocity (ba-PWV) values was used for analysis [24]. Hyperlipidemia related variables, Triglyceride (TG) was analyzed using an autoanalyzer (ADVIA 1650, Bayer, Japan) applying an enzymatic colorimetric method (intra-assay coefficients of variability (CV%): 1.2%, inter CV%: 1.4%), and total cholesterol (TC) was analyzed through a chemical reaction principle using cholesterol oxidase (intra CV%: 0.7%, inter CV%: 1.5%). High-density lipoprotein cholesterol (HDL-C) and low-density lipoprotein cholesterol (LDL-C) were analyzed using cholesterol oxidase and enzyme immunoassay, respectively (intra CV% and inter CV%: under 2.0%). Among diabetes related variables, the fasting plasma insulin (FPI) test was done using Insulin Kit (Roche, Germany) as an electrochemiluminescence immunoassay (ECLIA), while the fasting plasma glucose (FPG) was analyzed by enzyme assay using GLU Kit (Roche, Germany) and Modular Analytics (E170, Roche, Germany). The CVs were as follows: FPI: intra CV% 1.2, inter CV% 3.5%; FPG: intra CV% 0.4%, inter CV% 1.2%. The insulin resistance index, homeostasis model assessment-insulin resistance (HOMA-IR), were calculated by the formulae by Matthews *et al*. [25]. Among the markers of vascular inflammatory and insulin-like growth factor-1 (IGF-1), high sensitivity C-reactive protein (hs-CRP) was analyzed through particle enhanced immunothelometry using BNTM System (high sensitivity CRP, dade Behring, Marburg, Germany; intra CV%: 2.4%, inter CV%: 3.7%). IL-6 was analyzed using ELISA kit (ELISA, Biosource International, Camarillo, CA, USA) for cytokine measurement using plasma (intra CV%: 4.3%, inter CV%: 6.8%). IGF-1 was analyzed via chemiluminescent immunometric assay (CLIA) using Nichols Institute Diagnostics (US). IGF-1 assays was intra CV% 4.7% and inter CV% 6.6%.

### 2.4 Statistical Analysis

The mean and standard deviation for all dependent variables were calculated. Participants may be excluded from the entire analysis set if there is no data available after randomization according to the intent-to-treat (ITT) principle. In this study, ITT analysis was conducted excluding two dropouts (one control group and one test group) who had not completed all tests after registration, and the ITT analysis participants conducted in this study were consistent with the per protocol (PP) analysis participants. The Kolmogorov–Smirnov test was used to verify the normality of distribution for all outcome variables. A two-way analysis of variance (ANOVA) with repeated measures of the “time” factor was used to compare the EG and CG groups and to gage the effects of the 12-week exercise intervention for each dependent variable. The Bonferroni post-hoc testing method was used to identify within-group changes of time. Clinically meaningful changes were assessed by determining the mean change and 95% confidence interval (CI). All analyses were done using Statistical Package for the Social Sciences (SPSS) version 25.0 (IBM Corporation, Armonk, NY, USA). A priori, the level of significance was set at 0.05.

## 3. Results

The body composition parameters for both groups are shown in Table 4 and a significant interaction between the two groups was found for BMI (*p* = 0.029, η^2^ = 0.170), fat-free mass (*p* = 0.009, η^2^ = 0.232), % body fat (*p <* 0.001, η^2^ = 0.549), and WHR (*p* = 0.013, η^2^ = 0.217). Post-hoc analyses exhibited significant decreases in BMI (EG: –0.80 [–1.53, –0.06], *p *< 0.05), % body mass (EG: –2.87 [–4.04, –1.70] %, *p <* 0.001), and WHR (EG: –0.03 [–0.05, 0.00], *p <* 0.05) in EG. Fat-free mass (EG: 0.86 [0.27, 1.45] kg, *p <* 0.01) was significantly increased in the EG.

**Table 4.** **Before and after training data (mean ± SD) for body composition with main analysis of variance results**.

Measure	CG			EG			*$\eta^{2}$* (*p*) value		
	Before	After	Mean change	Before	After	Mean change	Group	Time	Interaction
			95% CI			95% CI			
Body weight	52.91 ±	52.97 ±	0.06	52.43 ±	50.75 ±	–1.68	0.022	0.121	0.136
(kg)	5.03	4.71	[–0.53, 0.64]	5.20	4.13	[–3.43, 0.08]	(0.449)	(0.069)	(0.053)
BMI	22.58 ±	22.61 ±	0.03	22.50 ±	21.70 ±	–0.8	0.030	0.149	0.170
(kg/$m^{2}$)	1.69	1.60	[–0.23, 0.29]	1.75	1.11	[–1.53, –0.06]*	(0.382)	(0.043)†	(0.029)†
Free fat mass (kg)	32.89 ±	32.80 ±	–0.08	32.51 ±	33.38 ±	0.86	0.000	0.169	0.232
	2.56	2.96	[–0.52, 0.35]	1.93	1.72	[0.27, 1.45]**	(0.912)	(0.030)†	(0.009)†
Fat mass	35.33 ±	36.04 ±	0.71	35.10 ±	32.23 ±	–2.87	0.105	0.307	0.549
(%)	3.18	3.19	[–0.01, 1.43]	3.13	3.23	[–4.04, –1.70]***	(0.093)	(0.002)†	(0.000)†
ASM,	5.17 ±	5.15 ±	–0.02	5.27 ±	5.32 ±	0.05	0.024	0.010	0.041
(kg/$m^{2}$)	0.59	0.55	[–0.13, 0.09]	0.26	0.26	[–0.04, 0.14]	(0.428)	(0.621)	(0.303)
WHR	0.93 ±	0.93 ±	0.01	0.93 ±	0.90 ±	–0.03	0.050	0.117	0.217
	0.04	0.04	[–0.01, 0.02]	0.06	0.05	[–0.05, 0.00]*	(0.253)	(0.075)	(0.013)†

Note: SD, standard deviation; CI, confidence interval; CG, control group; EG, exercise group; BMI, body mass index; ASM, appendicular skeletal mass; WHR, waist hip ratio. † Significant interaction or main effect. * *p *< 0.05; **p < 0.01; ***p < 0.001 vs. before training.

Cardiovascular risks factor parameters of pre-intervention and post-intervention data for both groups are shown in Table 5. Hypertension related variables included the significant interaction observed between the two groups in heart rate (*p* = 0.006, η^2^ = 0.260), SBP (*p* = 0.034, η^2^ = 0.162), and RPP (*p* = 0.001, η^2^ = 0.347). Post-hoc analyses established a significant decrease in heart rate (EG: –7.14 [–11.74, –2.54] beat/min, *p *< 0.01), SBP (EG: –7.43 [–12.94, –1.92] mmHg, *p *< 0.05), and RPP (EG: –1488.07 [–2279.37, –696.77], *p *< 0.01) in EG. Hyperlipidemia related variables included significant interaction observed between the two groups of HDL-C (*p* = 0.005, η^2^ = 0.271). Post-hoc analyses established significantly increase in HDL-C (EG: 6.16 [1.37, 10.95] mg/dL, *p *< 0.05) among EG. Atherosclerosis related variables included significant interaction in all values (TC/HDL-C ratio: *p *< 0.001, η^2^ = 0.395, TG/HDL-C ratio: *p* = 0.041, η^2^ = 0.151, LDL-C/HDL-C ratio: *p *< 0.001, η^2^ = 0. 378, ba-PWV: *p* = 0.024, η^2^ = 0.180). Post-hoc analyses established a significant decrease in TC/HDL-C ratio (EG: –0.71 [–1.13, –0.28], *p *< 0.01), TG/HDL-C ratio (EG: –0.72 [–1.31, –0.14], *p *< 0.05), LDL-C/HDL-C ratio (EG: –0.56 [–0.92, –0.20], *p *< 0.01), ba-PWV (EG: –65.29 [–127.37, –3.21] cm/s, *p *< 0.05) in EG. Diabetes mellitus related variables included significant interaction observed between the two groups of FPI (*p* = 0.013, η^2^ = 0.216) and HOMA-IR (*p* = 0.011, η^η^ = 0.225). Post-hoc analyses established significant improvements in FPI (EG: –1.73 [–2.85, –0.62], *p *< 0.01), and HOMA-IR (EG: –0.65 [–1.09, –0.21], *p *< 0.01) among EG.

**Table 5.** **Before and after training data (mean ± SD) for cardiovascular risk factors with main analysis of variance results**.

Measure	CG			EG			*$\eta^{2}$* (*p*) value		
	Before	After	Mean change	Before	After	Mean change	Group	Time	Interaction
			95% CI			95% CI			
Hypertension related variables									
Heart rate	75.93 ±	76.43 ±	0.50	75.64 ±	68.52 ±	–7.14	0.071	0.210	0.260
(beats·$\min^{-1}$)	10.10	9.31	[–2.43, 3.43]	6.71	7.09	[–11.74, –2.54]**	(0.172)	(0.014)†	(0.006)†
SBP	133.90 ±	133.43 ±	–0.50	132.57 ±	125.14 ±	–7.43	0.045	0.202	0.162
(mmHg)	13.05	9.50	[–4.27, 3.27]	15.56	9.73	[–12.94, –1.92]*	(0.278)	(0.016)†	(0.034)†
DBP	81.10 ±	80.31 ±	–0.79	80.29 ±	76.15 ±	–4.14	0.033	0.095	0.047
(mmHg)	9.97	8.40	[–3.00, 1.42]	6.43	6.68	[–10.17, 1.89]	(0.353)	(0.110)	(0.270)
MAP	98.70 ±	98.01 ±	–0.69	97.71 ±	93.65 ±	–5.23	0.044	0.145	0.091
(mmHg)	10.80	8.99	[–3.02, 1.64]	9.09	6.72	[–10.87, 0.40]	(0.286)	(0.046)†	(0.119)
PP	52.80 ±	53.09 ±	0.29	52.29 ±	48.99 ±	–3.29	0.024	0.064	0.089
(mmHg)	5.32	7.54	[–3.11, 3.69]	10.76	9.17	[–6.75, 0.17]	(0.436)	(0.192)	(0.123)
RPP	10191.4 ±	10208.6 ±	17.29	10090.3 ±	8602.2 ±	–1488.07	0.072	0.336	0.347
	1882.5	1525.4	[–357.53, 392.10]	1902.1	1380.0	[–2279.37, –696.77]**	(0.170)	(0.001)†	(0.001)†
Hyperlipidemia related variables									
TC	192.29 ±	193.71 ±	1.43	182.93 ±	172.07 ±	–10.86	0.088	0.031	0.051
(mg·${\mathrm{dL}}^{-1}$)	32.25	28.96	[–4.07, 6.92]	25.91	29.88	[–32.56, 10.84]	(0.126)	(0.371)	(0.246)
TG	116.93 ±	119.24 ±	2.31	107.57 ±	89.70 ±	–17.88	0.056	0.036	0.059
(mg·${\mathrm{dL}}^{-1}$)	62.91	50.90	[–24.78, 29.40]	34.01	30.5	[–38.86, 3.11]	(0.226)	(0.336)	(0.214)
LDL-C	118.47 ±	122.16 ±	3.69	111.27 ±	97.82 ±	–13.45	0.076	0.041	0.117
(mg·${\mathrm{dL}}^{-1}$)	36.46	30.09	[–1.24, 8.61]	22.38	33.4	[–32.77, 5.87]	(0.156)	(0.300)	(0.075)
HDL-C	49.71 ±	47.86 ±	–1.86	50.14 ±	56.30 ±	6.16	0.058	0.097	0.271
(mg·${\mathrm{dL}}^{-1}$)	8.05	8.65	[–4.71, 1.00]	13.48	8.36	[1.37, 10.95]*	(0.217)	(0.107)	(0.005)†
Atherosclerosis related variables									
TC/HDL-C ratio	3.99 ±	4.26 ±	0.27	3.87 ±	3.16 ±	–0.71	0.073	0.118	0.395
	1.27	1.38	[–0.02, 0.55]	1.09	0.82	[–1.13, –0.28]**	(0.164)	(0.073)	(0.000)†
TG/HDL-C ratio	2.61 ±	2.74 ±	0.13	2.38 ±	1.66 ±	–0.72	0.051	0.078	0.151
	2.00	1.93	[–0.50, 0.76]	1.21	0.73	[–1.31, –0.14]*	(0.250)	(0.150)	(0.041)†
LDL-C/HDL-C ratio	2.50 ±	2.71 ±	0.21	2.39 ±	1.83 ±	–0.56	0.073	0.113	0.378
	1.03	1.08	[0.00, 0.42]	0.87	0.76	[–0.92, –0.20]**	(0.165)	(0.080)	(0.000)†
ba-PWV	1808.4 ±	1844.9 ±	36.46	1798.7 ±	1733.4 ±	–65.29	0.017	0.026	0.180
(cm/s)	219.4	164.9	[–31.46, 104.38]	200.3	214.2	[–127.37, –3.21]*	(0.504)	(0.413)	(0.024)†
Diabetes mellitus related variables									
FPG	107.50 ±	108.46 ±	0.96	107.79 ±	101.04 ±	–6.75	0.010	0.075	0.126
(mg·${\mathrm{dL}}^{-1}$)	20.02	18.10	[–2.96, 4.89]	22.70	12.94	[–14.40, 0.90]	(0.605)	(0.158)	(0.063)
FPI	7.31 ±	7.20 ±	–0.011	6.82 ±	5.09 ±	–1.73	0.116	0.264	0.216
(μU·${\mathrm{mL}}^{-1}$)	2.27	1.82	[–0.80, 0.57]	2.30	1.65	[–2.85, –0.62]**	(0.076)	(0.005)†	(0.013)†
HOMA-IR	1.98 ±	1.95 ±	–0.03	1.92 ±	1.28 ±	–0.65	0.069	0.259	0.225
	0.77	0.69	[–0.24, 0.18]	0.95	0.50	[–1.09, –0.21]**	(0.176)	(0.006)†	(0.011)†

Note: SD, standard deviation; CI, confidence interval; CG, control group; EG, exercise group; SBP, systolic blood pressure; DBP, diastolic blood pressure; MAP, mean arterial pressure; PP, pulse pressure; RPP, rate pressure product; TC, total cholesterol; TG, triglyceride; LDL-C, low density lipoprotein cholesterol; HDL-C, high density lipoprotein cholesterol; ba-PWV, brachial ankle pulse wave velocity; FPG, fasting plasma glucose; FPI, fasting plasma insulin; HOMA-IR, homeostatic model assessment for insulin resistance. † Significant interaction or main effect. * *p *< 0.05; **p < 0.01; ***p < 0.001 vs. before training.

**Fig. 2.**
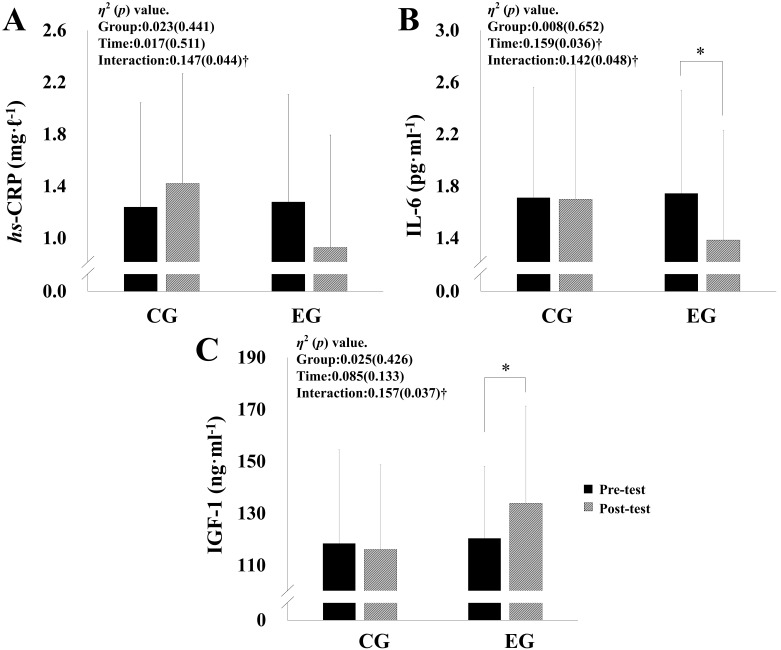
**Effects of vascular inflammatory markers, and insulin-like growth factor-1**. (A) High sensitivity C-reactive protein (hs-CRP). (B) Interleukin 6 (IL-6). (C) Insulin-like growth factor-1 (IGF-1). † Significant interaction or main effect. * *p *< 0.05 vs. before training.

Inflammatory markers and IGF-1 parameters of pre-intervention and post-intervention data for both groups are presented in Fig. 2. Inflammatory markers variables showed significant interaction among all values (hs-CRP: *p* = 0.044, η^2^ = 0.147, IL-6: *p* = 0.048, η^2^ = 0.142). Post-hoc analyses established a significant decrease IL-6 (EG: –0.036 [–0.68, –0.04], *p <* 0.05) in EG. IGF-1 showed significant interaction (IGF-1: *p* = 0.037, η^2^ = 0.157), and IGF-1 (EG: 13.50 [2.04, 24.95], *p <* 0.05) was significantly improved among the EG.

## 4. Discussion

In the 12-week circuit training conducted in this study, the average attendance rate was 98% or more, and one person each in the exercise group and the control group dropped out due to personal circumstances. As a side effect of circuit training in this study, there was some muscle pain at the beginning of exercise, but it was quickly recovered. Other than that, there were no specific side effects.

Recently, fitness training and circuit training for the elderly were selected as the world’s best health and fitness trends by the ACSM [18, 19]. This study attempted to investigate the effects of cardiovascular risk factors, vascular inflammatory markers, and IGF-1 on obese women with sarcopenia by conducting circuit training selected as the latest trend for 12 weeks. The results of this study showed a significant decrease in BMI, % body fat, WHR, and significantly increased fat-free mass among the exercise group, contrary to the control group. Generally, combined exercise was reported to induce both reduction of body fat through fat oxidation and increase in muscle mass owing to protein synthesis [26, 27]. There was little change in body weight as a result of combined exercise. However, it was consistent with the results of previous studies that a decrease in body fat and an increase in lean body mass were observed. In this study, it was suggested that the improvement in body composition was observed without significant changes in weight since the aerobic exercise effective against reducing body fat and the resistance exercise effective against increasing muscle mass was done by the elderly obese women with sarcopenia. Likewise, Vincent *et al*. [28] reported that combined exercise training aimed at improving sarcopenic obesity and improving body function is effective when training is conducted more than twice a week for at least three months. Based on the results of this study, where a positive effect on body composition was observed by applying combined exercise three times a week for 12 weeks, the period and frequency of circuit exercise conducted in this study was suitable for improving the body composition in women with sarcopenic obesity.

Hypertension is a common cause for excessive burden on the heart, which increases the incidence of cardiovascular disease [29]. SBP and DBP in addition to PP and MAP are also used as indicators for risk prediction of cardiovascular disease such as arteriosclerosis [30]. Particularly, RPP, which is calculated through the multiplication of SBP and HR, is an indicator of the burden on myocardial muscle, which suggests that low RPP is good for myocardial function and low ischemia [31]. In this regard, Park *et al*. [32] argued that the risk of hypertension in the elderly with sarcopenia is higher compared to that of the normal elderly, such that the elderly with sarcopenia should be more interested in preventing hypertension. This study result showed that DBP, PP, and MAP tended to decrease, while HRrest, SBP, and RPP were significantly reduced. The previous study reported a significant decrease in SBP, DBP, HRrest, and RPP after exercise [33, 34] but it is interpreted that only significant decrease in HRrest, SBP, and RPP in this study, and no significant decrease in blood pressure since it is within normal range. Batrakoulis* et al*. [35] studies that conducted 20-week circuit exercise training for overweight and obese women showed an improvement in average arterial pressure, which is believed to require longer-term exercise for variables related to blood pressure. However, given the significant changes in the RPP, which suggests there is a burden on myocardial muscles along with the decrease in HRrest and SBP, it can be concluded that the 12-week circuit conducted in this study is likely to result to an improvement in the cardiovascular functions among women with sarcopenic obesity.

Regular exercises have a positive effect on changes in hyperlipidemia, while long-term exercise regime is known to reduce TC, TG, and LDL-C and increase HDL-C [36]. However, in this study, TC, TG, and LDL-C were reduced, despite no significant changes made, and only HDL-C was significantly increased, resulting in rather different outcomes from the preceding study. In the preceding study, TC and LDL-C have been reported to be effectively reduced during the exercise periods, when they are high in exercise and involve weight loss [37]. In this study, it is construed that the lack of significant weight loss in exercise groups was limited to the changes in TC and LDL-C. Therefore, it is expected that further action involving higher-intensity exercise, as well as weight changes, will provide a clean-up in regards to problems such as changes in TC and LDL-C. Moreover, in this study, the HDL-C was significantly increased through the 12-week circulation exercise. In this regard, Tambalis *et al*. [36] reported an increase in HDL-C when exercising at least 75% of HRmax and is consistent with the strength of the motion conducted in this study. Consequently, the cyclical motion programs taken care of in this study are effective against the increase in HDL-C among women with sarcopenic obesity.

The 12-week circuit exercises on arteriosclerosis-related variables in elderly obesity women with sarcopenia was found to significantly reduce in all variants of the exercise group. High strength training is suggested to improve the arteriosclerosis index for the elderly [36, 38], however, given the results of a study that showed significant effects in low intensity in older adults with illnesses [39], the intensity of the exercise conducted in older adults with sarcopenia and obesity diseases as per the current study was deemed appropriate.

Exercise training, one of the methods of improving insulin resistance is very effective and acts by increasing the expression of free fatty acid oxidation and blood glucose transportation, thereby regulating blood glucose and enhance the insulin sensitivity [40, 41, 42]. In this regard, Mann *et al*. [43] reported that combined treatment through resistance and aerobic exercise was more effective in enhancing insulin resistance as compared to single exercise. In this study, there was no significant change in fasting blood glucose after exercise training, despite significant reduction in fasting insulin and HOMA-IR. This suggests that insulin sensitivity is improved through regular exercise, and so is the ability to control fasting blood glucose from a small amount of fasting insulin. Therefore, circuit exercise is believed to contribute to the prevention and improvement of diabetes in the elderly obese women with sarcopenia.

In this study, hs-CRP and IL-6 were significantly decreased in the exercise group after 12 weeks of circuit exercise. hs-CRP and IL-6 were found to have a positive correlation with body fat mass and a negative correlation to muscle mass, and two variables have been reported to be closely related to sarcopenic obesity [44]. This suggests that the improvement in body composition, such as the decrease in body fat and the increase in fat-free mass, is associated with the improvement of inflammatory indicators in this study. In addition, Beyer *et al*. [45] reported that the duration of exercise in the previous study, which showed significant changes in inflammatory markers through exercise training, varied from 10 weeks to 18 months, while the frequency varying from 1 to 3 times a week, with a moderate intensity. Therefore, the circuit exercise was found to be effective against inflammatory markers among the elderly obese women with sarcopenia.

IGF-1 was negatively correlated to % body fat, positively correlated to fat-free mass, and reported to decrease as a result of aging [46, 47]. Therefore, it is inferred that IGF-1 levels are low in sarcopenic obese elderly people with increased body fat and decreased muscle mass. The preceding studies examining the effects of exercise training on IGF-1 are as follows: In Chen *et al*. [48], IGF-1 significantly increased after 8 weeks of combined exercise. Annibalini *et al*. [49] also found out that IGF-1 significantly increased after 16 weeks of combined exercise. In the current study, a 12-week circuit exercise regime was conducted among elderly obese women with sarcopenia, and the IGF-1 increased significantly, which is consistent with the previous studies.

In summary, these results suggest that 12 weeks of circuit exercise could be presented as an effective way to improved sarcopenic obesity and enhance health through improvement of body composition, cardiovascular risk factors, inflammatory markers, and IGF-1 in elderly obese women with sarcopenia. This study had some limitations. We included only elderly women living in the community-dwelling. In addition, despite the positive effects on health-related variables and ASM values through circulation exercise, the subjects still fall under sarcopenia. In order to eliminate this limitation in the future, it is judged that it is necessary to combine nutritional intake such as protein intake or to conduct additional research with a longer-term and different intensity. It should also include men with sarcopenia and the elderly living in hospitals and nursing homes.

## 5. Conclusions

This study investigated the effects of a 12-week circuit training program on cardiovascular risk factors, vascular inflammatory markers, and IGF-1 in elderly obese women with sarcopenia. The key results obtained from this study are as follows: The BMI, fat-free mass, % body mass, and WHR were significantly improved in the exercise group. Among Cardiovascular risk factors, heart rate, SBP, RPP, HDL-C, TC/HDL-C ratio, TG/HDL-C ratio, LDL-C/HDL-C ratio, ba-PWV, fasting insulin, and HOMA-IR were significantly improved in the exercise group. hs-CRP and IL-6 had their inflammatory index significantly decreased among the exercise group participants, and IGF-1 was significantly increased. Therefore, the findings of this study indicate that circuit exercise has a positive effect on the improvement of cardiovascular risk factors, vascular inflammatory markers, and IGF-1 in elderly obese women with sarcopenia. Further research is required to investigate the effects of various types, intensity, time, frequency, and duration of exercise treatment on elderly people with sarcopenic obesity.
